# Association between masticatory ability and oral functions

**DOI:** 10.4317/jced.56747

**Published:** 2020-11-01

**Authors:** Mariko Maruyama, Koji Morita, Hitomi Kimura, Fumiko Nishio, Mitsuyoshi Yoshida, Kazuhiro Tsuga

**Affiliations:** 1 DDS, PhD, Clinical staff, Department of Advanced Prosthodontics, Graduate School of Biomedical & Health Sciences, Hiroshima University, Hiroshima, Japan; 2 DDS, PhD, Assistant professor, Department of Advanced Prosthodontics, Graduate School of Biomedical & Health Sciences, Hiroshima University, Hiroshima, Japan; 3 DDS, Clinical staff, Department of Advanced Prosthodontics, Graduate School of Biomedical & Health Sciences, Hiroshima University, Hiroshima, Japan; 4 DDS, PhD, Associated professor, Department of Advanced Prosthodontics, Graduate School of Biomedical & Health Sciences, Hiroshima University, Hiroshima, Japan; 5 DDS, PhD, Professor, Department of Advanced Prosthodontics, Graduate School of Biomedical & Health Sciences, Hiroshima University, Hiroshima, Japan

## Abstract

**Background:**

Mastication is the process of breaking ingested food with the teeth and mixing it with saliva to form a mass that is easy to swallow. However, few studies have reported on oral functions, such as occlusal force, tongue pressure, and mastication. The purpose of this study was to evaluate the association between masticatory function and oral functions, such as occlusal force and tongue pressure.

**Material and Methods:**

In this study, there were 113 patients (41 men and 72 women; mean age, 68.4 ± 11.3 years) who visited dentists at the Hiroshima University Hospital, Hiroshima, Japan between April 2015 and November 2018. Masticatory function of the patients was evaluated using a masticatory ability test system. In addition, occlusal force was measured using a pressure-sensitive film and the maximum tongue pressure was measured with a tongue pressure measuring device according to a conventional method. The relationship of masticatory ability with occlusal force and tongue pressure was examined using multivariate analysis while considering patients’ age, gender, and the number of remaining teeth.

**Results:**

Masticatory ability was significantly related to occlusal force, maximum tongue pressure, age, body mass index, the number of remaining teeth, and occlusal contact area (*p*< 0.05). Multiple regression analysis identified that masticatory ability was significantly associated (*p*< 0.05) with occlusal force and maximum tongue pressure.

**Conclusions:**

Masticatory ability was significantly associated with occlusal force and maximum tongue pressure, indicating that the large muscle mass in the oral cavity is indispensable for improving masticatory function.

** Key words:**Mastication, tongue pressure, occlusion force, oral function.

## Introduction

Mastication is defined as the grinding of the ingested food and mixing it with saliva to form a bolus which is easy to swallow (1). Mastication is performed not only by teeth, but also by the movement of perioral muscles (2,3). In other words, mastication involves various functions that are related to each other, such as feeding, biting, crushing, mixing, bolus formation, and swallowing. Since it is difficult to objectively and quantitatively evaluate each of these functions separately, the factors that influence the masticatory ability are still unclear. However, the masticatory ability to bite and crush can be objectively evaluated using the masticatory ability inspection system (4,5). The role of teeth and tongue in mastication and swallowing is indispensable. Most of the studies on masticatory function have focused on jaw movements up until now. However, it is extremely important to investigate the changes in the pressure exerted by the tongue to perform movements that form a bolus, and to objectively conduct masticatory function tests (6). In addition, oral function, including tongue movement, declines with aging (7). However, few studies have investigated the relationship between the masticatory ability that forms the bolus and oral functions such as occlusal force and tongue pressure generated by muscles around the oral cavity. Therefore, we hypothesize that masticatory function is related to oral functions and oral states. The purpose of this study was to evaluate the relationship between masticatory ability and various oral functions and oral states.

## Material and Methods

-Subjects

The study subjects were 113 patients (41 men, 72 women; mean age, 68.4 ± 11.3 years) who visited the Hiroshima University Hospital, Hiroshima, Japan from April 2015 through November 2018. Individuals with severe periodontitis, cerebrovascular disorders, and neuromuscular disorders that affected masticatory ability were excluded from the study. 19 individuals who wear complete dentures and 81 individuals who wear fixed or removable dentures were included in this study. The purpose of the study, methods, and expected results were explained to each subject, and those who agreed to participate and signed the informed consent form were included in the study. This study was conducted with the approval of the Hiroshima University Epidemiological Research Ethics Review Board (approval number: E-920-1).

-Measurement method

A self-administered questionnaire was used to record a subject’s age, and sex. Body mass index (BMI), number of remaining teeth, and temporomandibular disorders were measured by a dentist with more than 10 years of dental treatment experience. In addition, tests were performed to evaluate the masticatory ability, occlusal force, and tongue pressure.

Masticatory ability: Masticatory ability system is a method of measuring the chewing ability from glucose contained in gummy when a subject performs mastication prescribed gum in a certain period of time. A test gummy jelly with standardized ingredients and shape and a glucosensor was used to investigate the masticatory ability. Immediately after the subject was allowed to chew the gummy jelly freely for 20 seconds, the whole bolus was rinsed with 10 ml of water and the subject spat into a filtration mesh. Glucose was eluted from the bitten gummy jelly into water and masticatory ability was calculated from the glucose concentration (8).

Occlusal force: The occlusal force measurement at the intercuspal position was recorded while keeping the subject’s occlusal plane horizontal. First, the subject occluded into Dental Prescale, a pressure-sensitive film (Dental Prescale, GC, Tokyo), applying maximum occlusal force for about 3 seconds. Second, the portions of the film that developed color changes under pressure were analyzed using an occlusal force measurement system (Occluzer, GC, Tokyo) and the occlusal contact area and occlusal force were calculated.

Tongue pressure: The maximum tongue pressure was measured using a tongue pressure measuring device (TPM-01, JMS, Hiroshima) by pressing the balloon part of the probe over the tongue for 7 seconds at the maximum voluntary effort.

-Statistical analysis

The relationship between masticatory ability as the dependent variable and all the explanatory variables was examined using the Spearman’s rank correlation coefficient (ρ). In addition, the explanatory variables were tested using the Spearman’s rank correlation coefficient and highly significant (*p* < 0.001) variables were excluded due to multicollinearity. Multiple regression analysis was performed using the remaining variables to identify the factors affecting masticatory ability. A *p*-value less than 0.05 was considered statistically significant.

## Results

Means and standard deviations of all the variables are shown in Table 1. The results of bivariate analysis of masticatory ability as the dependent variable and all the explanatory variables are shown in Table 2. Spearman’s rank correlation coefficient showed that masticatory ability was significantly associated with age (ρ: – 0.25, *p* < 0.001), BMI (ρ: 0.2, *p* < 0.05), number of remaining teeth (ρ: 0.48, *p* < 0.001), occlusal force (ρ: 0.54; *p* < 0.001), occlusal contact area (ρ: 0.5, *p* < 0.001), and tongue pressure (ρ: 0.3, *p* < 0.001). Additionally, the number of remaining teeth was strongly correlated with age (ρ: – 0.44, *p* < 0.001), occlusal force (ρ: 0.38, *p* < 0.001), and occlusal contact area (ρ: 0.42, *p* < 0.001); age was correlated with temporomandibular disorders (ρ: – 0.22, *p* < 0.05), clenching (ρ: – 0.2, *p* < 0.05), and tongue pressure (ρ: – 0.23, *p* < 0.05); BMI was correlated with tongue pressure (ρ: 0.26, *p* < 0.05); and occlusal force was correlated with the occlusal contact area (ρ: 0.88; *p* < 0.001). Consequently, the number of remaining teeth, age, BMI, and occlusal contact area were excluded from the multiple regression analysis due to multicollinearity. The multiple regression analysis using a stepwise approach in Table 3 showed that masticatory ability was significantly associated with occlusal force and maximum tongue pressure (*p* < 0.05).

**Table 1:**
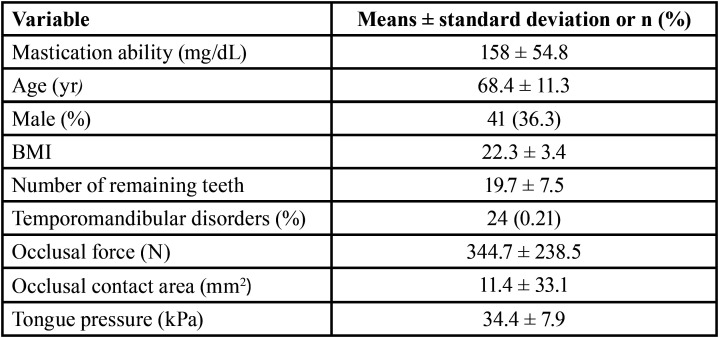
Mean and standard deviation of each variable.

**Table 2:**
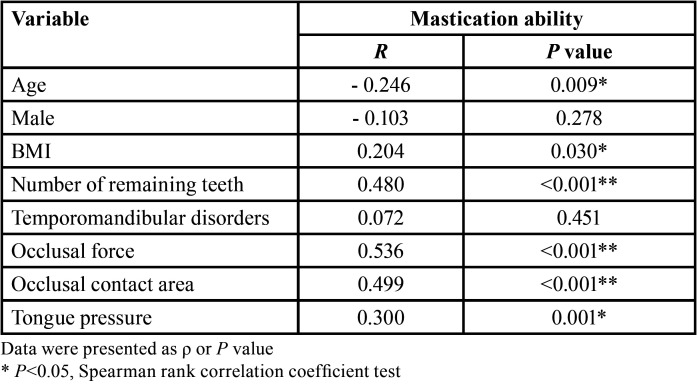
Spearman’s rank correlation coefficient for the association with outcome variable and predictor variables.

**Table 3:**
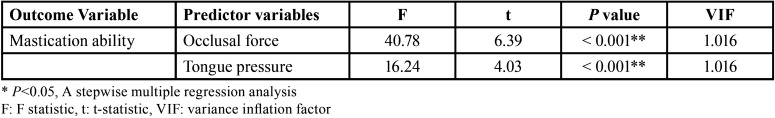
Multiple Regression Analysis.

## Discussion

The results of this study showed that masticatory ability was significantly associated with occlusal force and tongue pressure.

Our findings are similar to previous studies that reported that masticatory ability was significantly related to occlusal pressure (an indicator of occlusal force) and oral diadochokinesis (an indicator of tongue movement). In addition, a study reported that masticatory ability decreases with decreasing occlusal force due to a reduction in occlusal support area (9). In their study, masticatory ability was not related to the number of remaining teeth, whereas the results of Spearman’s rank correlation coefficient in our study showed a significant correlation between occlusal force and the number of remaining teeth. These results might not have been significantly different since the mastic elasticity in this study was measured with the occlusal support zone restored with a removable or fixed denture to replace missing teeth.

Tongue pressure has been noted as an objective index of oral function in eating disorders and dysphagia (10). The tongue skillfully changes its position and shape during mastication due to the advanced neural mechanism (11), and its movement dexterity plays an important role in the expression of masticatory function (12). Tongue pressure is associated with tongue muscle mass (13) and tongue movement (14). From these reports, the result that the masticatory ability was related to tongue pressure in the present study indicates that the movement itself to form a bolus which is easy to swallow by crushing the ingested food with teeth and mixing it with saliva is important. As in previous reports, our results suggest that chewing does not take place separately from swallowing, but the two processes proceed in a coordinated manner (15). Masticatory ability, occlusal force, and tongue pressure are also important factors for oral dysfunction (10), and it is further evident from our study that maintenance of these functions plays an important role in preventing oral dysfunction.

This study has some limitations. Only healthy older subjects were enrolled in this study. The elderly who were in need of nursing and whose tongue pressure and movement were hindered were not included. In order to clarify the importance of tongue movement in mastication, a research plan for the elderly requiring long-term care will be needed in the future. In addition, the masticatory ability test in this study mainly evaluated chewing and it is necessary to evaluate the ability to mix the bolus as well.

## Conclusions

Masticatory ability was significantly related to occlusal force and maximum tongue pressure, indicating that the large muscle mass in the oral cavity is indispensable for improving masticatory function.
